# A deep learning-based automated image analysis for histological evaluation of broiler pectoral muscle

**DOI:** 10.1016/j.psj.2023.102792

**Published:** 2023-05-19

**Authors:** Jonathan Dayan, Noam Goldman, Daniel Waiger, Tal Melkman-Zehavi, Orna Halevy, Zehava Uni

**Affiliations:** ⁎ Department of Animal Science, The Robert H. Smith Faculty of Agriculture, Food and Environment, The Hebrew University of Jerusalem, Rehovot 7610001, Israel; † Koret School of Veterinary Medicine, The Robert H. Smith Faculty of Agriculture, Food and Environment, The Hebrew University of Jerusalem, Rehovot 7610001, Israel; ‡ Center for Scientific Imaging, The Robert H. Smith Faculty of Agriculture, Food and Environment, The Hebrew University of Jerusalem, Rehovot 7610001, Israel

**Keywords:** automated image analysis, histology, breast muscle, broiler, chicken

## Abstract

Global market demand for chicken breast muscle with high yield and quality, together with the high incidence rate of breast muscle abnormalities in recent years highlights the need for tools that can provide a rapid and precise evaluation of breast muscle development and morphology. In this study, we used a novel deep learning-based automated image analysis workflow combining Fiji (ImageJ) with Cellpose and MorphoLibJ plugins to generate an automated diameter and cross-sectional area quantification for broiler breast muscle. We compared data of myofiber diameter from 14-day-old broiler chicks, generated either by manual analysis or by automated analysis. Comparison between manual and automated analysis methods exhibited a striking accuracy rate of up to 99.91%. Moreover, the automated analysis method was much faster. When the automated analysis method was implemented on 84 breast muscle cross-section images it characterized 59,128 myofibers within 4.2 h, while manual analysis of 27 breast muscle cross-section images enabled analysis of 17,333 myofibers in 54 h. The automated image analysis method was also more productive, producing data sets of both diameter and cross-sectional area at an 80-fold higher rate than the manual analysis (26,279 vs. 321 data sets per hour, respectively). In order to demonstrate the ability of this automated image analysis tool to detect differences in breast muscle histomorphology, we applied it on cross sections from chicks of control and in ovo feeding group, injected with a methionine source [2-hydroxy-4-(methylthio) butanoic calcium salt (**HMTBa**)], known to effect skeletal muscle histomorphology. Analysis was performed on 19,807 myofibers from the control group and 21,755 myofibers from the HMTBa group and was completed in less than 1 h. The clear advantages of this automated image analysis workflow characterized by high precision, high speed, and high productiveness demonstrate its potential to be implemented as a reproducible and readily adaptable research or diagnostic tool for chicken breast muscle development and morphology.

## INTRODUCTION

In order to meet the constant increase in market demand for chicken meat, broilers have undergone intense selection for higher growth rate, feed conversion and meat yield (Havenstein et al., 2003a,b; Zuidhof et al., 2014). Moreover, market demand stresses the need for broilers with high breast muscle development and quality (Fletcher, 2004; Petracci et al., 2015). Thus, diagnostic and research tools that can provide a rapid and precise evaluation of breast muscle development and morphology are of high importance. This is further highlighted as in recent years the poultry industry is facing a dramatic increase in the prevalence of breast muscle abnormalities whose onset is of complex etiology and is not yet entirely clear (Soglia et al., 2021).

Muscle development begins during embryogenesis with the formation of myoblasts that proliferate and increase in numbers (undergo hyperplasia). During differentiation, myoblasts fuse to create multinucleated myotubes and myofibers. At hatch, the number of myofibers is determined and remains relatively constant (Halevy and Velleman, 2022). Thereafter, muscle growth is due to the proliferation, differentiation, and fusion of satellite cells (adult muscle stem cells) with existing myofibers thereby increasing myofiber size (hypertrophy). Thus, measurements of myofiber number and diameter (lesser diameter) (Dubowitz, 1985; Halevy et al., 2004) as well as fiber cross-sectional area enable us to use stained muscle sections to follow changes during muscle development. In the evaluation of muscle histomorphology, the number, diameter, and cross-sectional area of myofibers contained in stained muscle sections are determined. Traditionally, manual quantification of these characteristics required time-consuming and laborious manual methods, limiting the number of myofibers analyzed to only a few thousands.

Lately, novel automated image analysis tools have become available, improving the capacity to evaluate data within a significantly shorter time (Kostrominova et al., 2013; Smith and Barton, 2014; Wen et al., 2017; Kastenschmid et al., 2019; Reyes-Fernandez et al., 2019). Human diseases involving repetitive cycles of muscle degeneration and regeneration, leading to muscle damage (e.g., Duchenne muscular dystrophy), have been an important driving force in the development of these tools (Rahmati and Rashno, 2021; Konnaris et al., 2022). Automatic methods for fiber geometry analysis were shown to operate with similar accuracy and less user-introduced variability to manual quantification. Although there are several histological methods to assess skeletal muscle degeneration and regeneration (Dubuisson et al., 2022), almost all were developed for mice and humans with myofibers that are considered relatively uniform compared to the highly variable terrain of broiler skeletal muscle. To our knowledge, we are the first to introduce an automated image analysis workflow designed for broiler skeletal muscle, providing a rapid and high-precision histological evaluation of tens of thousands of myofibers.

In this study, we use a deep learning-based automated image analysis workflow combining Fiji (ImageJ) with Cellpose and MorphoLibJ plugins to generate an automated diameter and cross-sectional area quantification for avian skeletal muscle. We compare data of myofiber diameter from 14-day-old broiler chicks, generated via manual or automated analysis, demonstrating that our automated analysis tool has a high level of accuracy and productivity, namely, the capacity to rapidly analyze data. This rapid analysis method is easily reproducible and readily adaptable. In order to demonstrate the ability of this automated image analysis tool, we examined myofiber geometry of 10-day-old control chicks to chicks which were in ovo fed with methionine source [2-hydroxy-4-(methylthio) butanoic calcium salt (**HMTBa**)]. Methionine was chosen based on its potential to effect skeletal muscle histomorphology, as shown in cattle and fish (Hosford et al., 2015; Fang et al., 2021).

## MATERIALS AND METHODS

### Birds

#### Stage 1: Comparison Between Manual and Automated Analysis Methods

Fertile eggs (*n* = 40; mean weight = 62.46 g, SD = 4.4 g) from 33-wk-old broiler hens (Cobb 500) were purchased from a commercial breeder farm (Y. Brown and Sons Ltd., Hod Hasharon, Israel). Eggs were incubated in a Petersime hatchery at the Faculty of Agriculture of the Hebrew University under standard conditions (37.8°C and 56% relative humidity). On embryonic (**E**) d 10, eggs were candled, and unfertilized eggs or dead embryo eggs were removed. At hatch, male chicks were transferred to brooders at the Faculty of Agriculture of the Hebrew University and reared according to the breeder recommendations (Cobb-Vantress). During the rearing period, chicks were fed with a standard commercial starter diet (formulated by Brown Feed Mill, Kaniel, Israel) with ad libitum access to water and feed. Tissue samplings for histological procedures were conducted on d 14 on 12 chicks. Stage 1 analysis included comparison between the manual analysis and automated analysis methods, using 27 images (cross sections of 9 birds, 3 images per cross section). Based on the accuracy of the results, an automated analysis of 84 images was performed (cross sections of 12 birds with 7 images per cross section).

#### Stage 2: Application of Automated Analysis With Control and HMTBa Treatments

Fertile eggs (*n* = 30; mean weight = 68.35 g, SD = 2.84 g) from 39-wk-old broiler hens (Cobb 500) were purchased from a commercial breeder farm (Y. Brown and Sons Ltd., Hod Hasharon, Israel). Incubation procedures were the same as described in stage 1. On E17.5, amniotic fluid (amnion) enrichment by in ovo feeding (**IOF**) was performed according to the procedure developed by Uni and Ferket (2003). Eggs were divided into 2 treatment groups; the HMTBa group was injected with 0.6 mL IOF solution of 3.5% 2-hydroxy-4-(methylthio) butanoic calcium salt (OH-Met; Adisseo France S.A.S., Commentry, France), while the control remained noninjected. IOF was performed using a 21-gauge needle, following a site of injection (**SOI**) pretest designed to verify that the IOF solution reaches the amnion successfully. After the IOF procedure was completed, all eggs were transferred to hatching trays. At hatch, male chicks were transferred to brooders at the Faculty of Agriculture of the Hebrew University and reared according to the breeder recommendations (Cobb-Vantress). During the rearing period, chicks were fed with a standard commercial starter diet (formulated by Brown Feed Mill, Kaniel, Israel) with ad libitum access to feed and water. Tissue samplings for histological procedures were conducted on d 10 on 4 chicks per treatment.

The animal study was reviewed and approved by IACUC: AG-20-16298.

### Muscle Sampling and Histological Procedures

Muscle samplings were performed as described by Halevy et al. (2004). In brief, muscle samples (approximately 0.5 cm × 0.5 cm × 1 cm) were removed from the superficial region of the proximal half of the left pectoralis major muscle. The muscle samples were fixed in 3.7% formaldehyde in PBS at pH 7.4 (Sigma-Aldrich, Rehovot, Israel) for 24 h. Then, samples were dehydrated, cleared, and embedded in paraffin. Cross sections of 4- to 6-µm thick were cut Leica RM2135 Microtome (Leica Biosystems, Nussloch GmbH, Germany), deparaffinized in Histochoice clearing agent (Sigma-Aldrich, St. Louis, MO), rehydrated, and stained with Picrosirius Red Fast Green (**SRFG**) staining to differentiate between myofibers (stained in green) and connective tissue (stained in red). After drying, samples were mounted with cover glass using DPX slide mounting medium (Sigma-Aldrich, St. Louis, MO). Finally, images [Tagged Image File Format (**Tiff**)] were visualized and generated using EVOS FL Auto-inverted microscope and are comprised of 12 stitched fields of ×60 magnification (µm to pixel ratio of 6.6).

### Image Analysis Workflow

Manual quantification was performed according to Halevy et al. (2004) using a standard Fiji-ImageJ (version 1.53t) measurement tool. Myofiber diameters were determined by analyzing the lesser diameter values as described by Dubowitz (1985).

**Figure 1:**
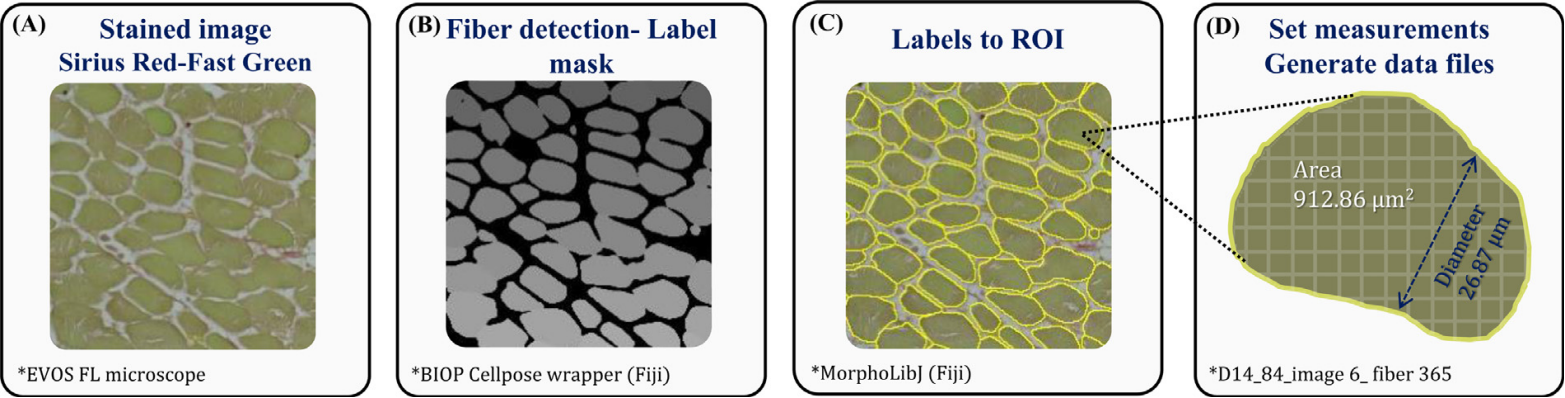
Automated image analysis workflow. (A) Raw images comprised of 12 stitched fields of ×60 magnification were generated using EVOS FL Auto-inverted microscope. Image processing included: (B) detection of the myofibers with BIOP Cellpose wrapper plugin; (C) conversion of the label masks to regions of interest (ROIs) with the MorphoLibJ plugin. The morphological analysis included the extraction of myofiber metrics. (D) The lesser diameter (μm) and cross-sectional area (μm^2^) were exported and saved to Excel files for further analysis and statistics.

The automated image analysis workflow for Fiji (https://doi.org/10.5281/zenodo.7678528) is based on 1) PT-BIOP and Cellpose wrapper plugins [Stringer et al., 2021; BIOP/ijl-utilities-wrappers: Wrappers for external software calls (Cellpose, Elastix, Ilastix...) and java utilities (object conversions and display) (github.com)], and 2) MorphoLibJ plugin (Legland et al., 2016). The script and code are available as a zenodo repository (https://doi.org/10.5281/zenodo.7678527). The workflow includes image capturing and analysis, as demonstrated in Figure 1. Image processing included the detection of the myofibers with BIOP Cellpose wrapper plugin and the conversion of the label masks to regions of interest (**ROIs**) with the MorphoLibJ plugin. The morphological analysis included the extraction of myofiber metrics; the lesser diameter (µm) and cross-sectional area (µm^2^) were exported and saved to Excel files for further analysis and statistics.

### Accuracy Rate, Variance, Productivity, and Statistics

For stage 1, in order to evaluate the similarity of results obtained by the automated analysis to results obtained by the manual analysis, the coefficient of variance (**CV**) and the Accuracy rate were calculated according to Kastenschmid et al. (2019). The CV is defined as: $\text{CV}=(\frac{\text{Standard}\;\text{Deviation}}{\text{Average}\;\text{value}})\times \;100$. The accuracy rate is defined as: $\text{Accuracy}=(1-\frac{\left|\text{Automated}\;\text{analysis}\;\text{value}-\text{Manual}\;\text{analysis}\;\text{value}\right|}{\text{Manual}\;\text{analysis}\;\text{value}})\times \;100$.

The Productivity rate is defined as the number of data sets generated per hour of work in each analysis method: $\text{Productivity}=\frac{\text{Amount}\;\text{of}\;\text{data}\;\text{generated}}{\text{Total}\;\text{time}\;\text{for}\;\text{analysis}\;}\;$.

For stage 2, statistical analysis of the data generated by automated image analysis was subjected to 1-way ANOVA followed by a Student *t* test and considered significantly different with a *P* value lower than or equal to 0.05 (*P* ≤ 0.05). All values are presented as mean size (μm) ± standard error mean (**SEM**). Statistical analysis was carried out using JMP-pro 16 software (SAS Institute Inc., Cary, NC).

## RESULTS AND DISCUSSION

Traditionally, the histomorphological evaluation of muscle cross sections required time-consuming and laborious manual methods. Recent developments in automated tools have been found to produce reliable analysis within a significantly shorter amount of time (Kostrominova et al., 2013; Smith and Barton, 2014; Wen et al., 2017; Kastenschmid et al., 2019; Reyes-Fernandez et al., 2019). However, these methods were developed mainly for mice and humans with skeletal muscle myofibers that are considered relatively uniform compared to the highly variable terrain of broiler skeletal muscle. Furthermore, as the poultry industry is facing a dramatic increase in the prevalence of breast muscle abnormalities and substantial economic loss (Soglia et al., 2021; Che et al., 2022), the need for novel research and diagnostic tools is highlighted. In this study, we present a deep learning-based automated image analysis workflow designed to provide a rapid and high-precision histological evaluation of broiler skeletal muscle.

### Stage 1: Comparison Between Manual and Automated Analysis Methods

**Figure 2:**
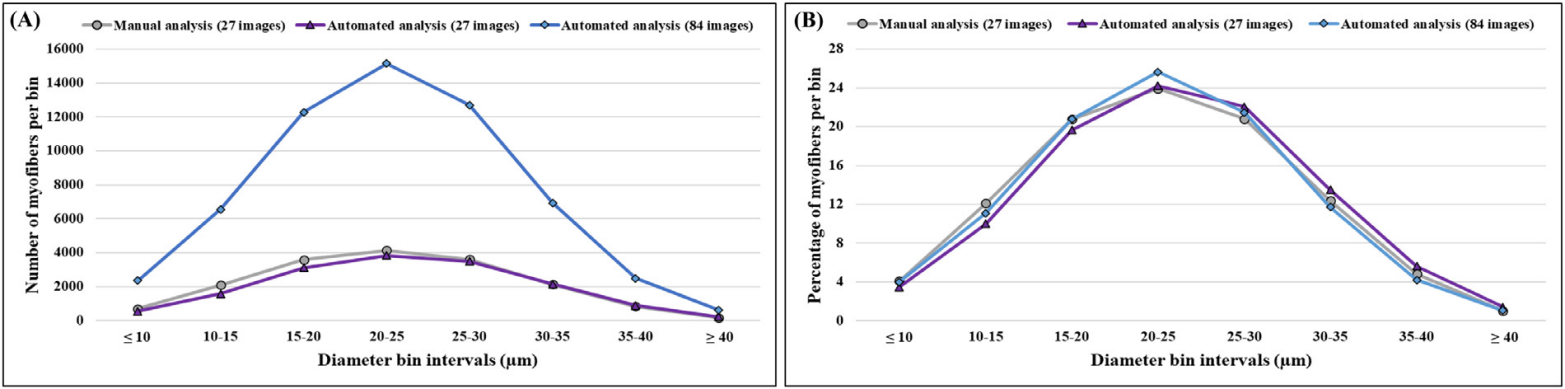
Myofiber diameter distribution in the pectoral muscle of 14-day-old broiler from manual and automated image analysis methods. Myofibers are clustered in bin intervals of 5 μm where (A) presents the diameter distribution of the actual number of counted myofibers and (B) presents the diameter distribution as percentage of the total myofibers. The images are comprised of 12 stitched fields of ×60 magnification and were generated using EVOS FL Auto-inverted microscope. The manual measurements were performed using the standard ImageJ measurement tool, and the automated measurements were performed using Fiji ImageJ with Cellpose and MorphoLibJ plugins.

In this study, we performed a comparison between the traditional method of manual measurement of myofiber diameter and results generated via automated image analysis using Fiji ImageJ software with Cellpose and MorphoLibJ plugins (illustrated in Figure 1). The data collected from 14-day-old broiler chicks and included 27 images (cross sections of 9 birds, 3 images per cross section) were analyzed in both manual and automated methods. Our findings show that the accuracy rate of the automated analysis is comparable to the manual analysis (96.84%) and therefore is highly accurate. Hence, we decided to further test the automated tool with a larger data set of 84 images (cross sections of 12 birds with 7 images per cross section). This enabled us to analyze a higher sample size, covering each cross section almost entirely (Table 1 and Figure 2).

**Table 1:** Comparison of image analysis methods.

Method	Number of myofibers	aMyofiber diameter (µm)	bCV	cAccuracy rate (%)	dMyofiber area (µm^2^)	Analysis time per scan (min)	Total time per analysis (h)	^e^Productivity per hour
Manual analysis (27 images)	17,333	22.86 ± 0.058	33.17	-	Not available	120	54	321
Automated analysis (27 images)	15,881	23.58 ± 0.059	31.99	96.84	717.69 ± 3.02	3	1.4	22,687
Automated analysis (84 images)	59,128	22.84 ± 0.031	32.21	99.91	676.44 ± 1.51	3	4.2	26,279

a Coefficient of variance (CV) was calculated for each analysis method as follows; $\text{CV}=(\frac{\text{Standard}\;\text{Deviation}}{\text{Average}\;\text{value}})\times \;100$.

b Accuracy was calculated compared to manual analysis as follows; $\text{Accuracy}=(1-\frac{\left|\text{Automated}\;\text{analysis}\;\text{value}-\text{Manual}\;\text{analysis}\;\text{value}\right|}{\text{Manual}\;\text{analysis}\;\text{value}})\times \;100$.

c Myofiber cross-sectional area is presented as mean size (µm^2^) ± standard error mean. Data are available only through automated analysis.

d Productivity (number of data sets generated per hour) was calculated for each analysis method as follows; $\text{Productivity}=\frac{\text{Amount}\;\text{of}\;\text{data}\;\text{generated}}{\text{Total}\;\text{time}\;\text{for}\;\text{analysis}\;}$.

The manual analysis of 27 images generated diameter measurements of 17,333 myofibers with an average size of 22.86 µm and variance of 33.17%, while the automated analysis of the same 27 images generated diameter measurements of 15,881 myofibers with an average size of 23.58 µm and variance of 31.99% with an accuracy rate of 96.84% (Table 1). The similarity in the variance and the high accuracy rate are a reassuring proof that the automated analysis method can generate results that are as accurate and precise as the traditional manual histological analysis. Furthermore, when a larger sample size of 84 images was examined, the automated analysis provided a striking accuracy rate of 99.91% with diameter measurements of 59,128 myofibers, average size of 22.84 µm and variance of 32.21% (Table 1). Overall, both manual and automated analysis methods provided results that are similar to previous publications, with myofiber-diameter distribution exhibiting the typical Gaussian curve (Halevy et al., 2006; Piestun et al., 2009, 2011, 2017; Patael et al., 2019), further supporting the accuracy of our automated analysis tool. Figure 2A shows the myofiber diameter distribution and the nearly identical plots from both manual and automated analysis, where the highest percentage of myofibers ranges between 20 and 25 µm (Figure 2B). In addition to the measurements of myofiber diameters, our automated analysis tool enabled us to obtain the cross-sectional area of each myofiber. Results from both methods show high similarity (93.9% accuracy) with an average value of 676.44 µm^2^ from the automated analysis of 84 images and an average value of 717.69 µm^2^ in the automated analysis of 27 images (Table 1).

The most prominent advantage of our automated analysis tool is the rapid analysis of a larger sample size (see time and productivity rate values in Table 1). While the manual analysis of each image takes 120 min, the automated analysis takes only 3 min. In addition, the overall analysis time in the manual method was 54 h, while the automated analysis was 1.4 h and 4.2 h for the 27 and 84 images, respectively. The automated analysis of 84 images enabled us to measure 59,128 fibers, more than triple the number of myofibers measured with the manual analysis in this experiment (17,333). Additionally, the number is much higher compared to previous studies which examined only few thousands (Halevy et al., 2006; Piestun et al., 2009, 2011, 2017; Patael et al., 2019).

Furthermore, we calculated the productivity rate for the automated and manual image analysis methods. The automated analysis of 27 and 84 images resulted in an astonishing number of 22,687 and 26,279 data sets (myofiber diameter and area) generated per hour, respectively. These productivity rates are 70- to 80-fold higher than the manual analysis, which generated only 321 data sets per hour.

The clear advantage of using automated analysis led us to evaluate this method and analyze the histomorphology of 10-day-old control chicks and IOF HMTBa-treated chicks. The automated tool enabled us to perform an analysis of a relatively large sample size with broad cross-sectional coverage. This analysis produced a better representation of the examined samples compared to the manual analysis while providing rapid and high-precision results.

### Stage 2: Application of Automated Analysis With Control and HMTBa Treatments

**Figure 3:**
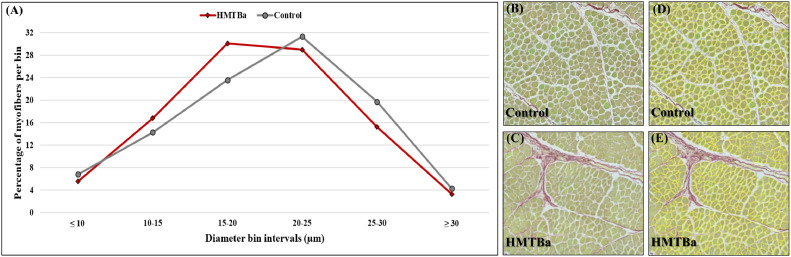
Myofiber diameter distribution in the pectoral muscle of 10-day-old broiler. Myofibers are clustered in bin intervals of 5 μm; (A) diameter distribution in percentage of total myofibers from 2 treatment groups, that is, control and HMTBa. (B, C) show 1 representative field of original sirius red fast green stained images from control and HMTBa treatments. Images are generated using EVOS FL Auto-inverted microscope with ×60 magnification and are comprised of 12 stitched fields; (D) and (E) myofiber identification by automated image analysis measurements performed using Fiji ImageJ with Cellpose and MorphoLibJ plugins.

Here we evaluated the histological parameters of the diameter and the surface area from 2 treatments, control and HMTBa (Figure 3B–E). Results were generated in less than 1 h and were performed on 19,807 myofibers from the control and 21,755 myofibers from the HMTBa treatment. The control group had significantly larger myofibers with an average diameter of 15.9 µm and area of 325.99 µm^2^, compared to an average diameter of 15.14 µm and area of 309.72 µm^2^ from the HMTBa treatment (Table 2). From these data we calculated the number of myofibers per area of 1 mm^2^ of muscle tissue. Although not significant, HMTBa had a higher number of myofibers per area than the control, with a value of 324.03 and 306.76 per 1 mm^2^, respectively. These values are reflected in the shift in myofiber diameter distribution (Figure 3A), where the HMTBa IOF treatment resulted in the highest percentage of myofibers ranging between 15 and 20 µm compared to 20-25 µm from the control experiment.

**Table 2:** Summary of morphological analysis and comparison between control and HMTBa treatments.

Treatment	Number of myofibers	Myofiber diameter (µm)	Myofiber area (µm^2^)	Number of myofibers/mm^2^
Control	19,807	15.9 ± 0.04*	325.99 ± 1.34*	306.76 ± 10.88
HMTBa	21,755	15.14 ± 0.036	309.72 ± 1.28	324.03 ± 13.47

Summary of morphological analysis and comparison between 2 treatment groups (control and HMTBa) in the pectoral muscle of a 10-day-old broiler. Myofiber diameter and area are presented as mean size ± standard error mean. The number of myofibers per mm^2^ was calculated for each image and normalized to total section area. Asterisk denotes the values that are significantly different between treatments, as derived from a 1-way ANOVA followed by Student *t* test (*P* ≤ 0.0001), *n* = 16 (4 birds per treatment and 4 images per bird). Images comprised of 12 stitched fields of ×60 magnification were generated using EVOS FL Auto-inverted microscope.

## CONCLUSIONS

In this study, we present a novel tool for histomorphology evaluation of broiler breast muscle using a deep learning-based automated image analysis workflow. When compared to the traditional manual method, the automated method shows many clear advantages, such as high precision, high speed, and high productivity rate. This rapid analysis method has the potential to be implemented as a readily adaptable research or diagnostic tool to evaluate chicken breast muscle development and morphology.

## DISCLOSURES

The authors declare no conflicts of interest.

## References

[bib0001] Che S., Wang C., Varga C., Barbut S., Susta L. (2022). Prevalence of breast muscle myopathies (Spaghetti meat, woody breast, white striping) and associated risk factors in broiler chickens from Ontario Canada. PLoS One.

[bib0002] Dubowitz V. (1985).

[bib0003] Dubuisson N., Versele R., Planchon C., Selvais C.M., Noel L., Abou-Samra M., Davis-López de Carrizosa M.A. (2022). Histological methods to assess skeletal muscle degeneration and regeneration in Duchenne muscular dystrophy. Int. J. Mol. Sci..

[bib0004] Fang C.C., Feng L., Jiang W.D., Wu P., Liu Y., Kuang S.Y., Tang L., Liu X.A., Zhou X.Q. (2021). Effects of dietary methionine on growth performance, muscle nutritive deposition, muscle fibre growth and type I collagen synthesis of on-growing grass carp (Ctenopharyngodon idella). Br. J. Nutr..

[bib0005] Fletcher D.L., Mead G.C. (2004). Pages 108–134 in Poultry Meat Processing and Quality.

[bib0006] Halevy O., Piestun Y., Allouh M.Z., Rosser B.W., Rinkevich Y., Reshef R., Rozenboim I., Wleklinski-Lee M., Yablonka-Reuveni Z. (2004). Pattern of Pax7 expression during myogenesis in the post hatch chicken establishes a model for satellite cell differentiation and renewal. Dev. Dyn..

[bib0007] Halevy O., Piestun Y., Rozenboim I., Yablonka-Reuveni Z. (2006). In ovo exposure to monochromatic green light promotes skeletal muscle cell proliferation and affects myofiber growth in post hatch chicks. Am. J. Physiol.-Regul. Integr. Compar. Physiol..

[bib0008] Halevy O., Velleman S.G., Scanes C.G., Dridi S. (2022). Sturkie’s Avian Physiology.

[bib0009] Havenstein G.B., Ferket P.R., Qureshi M.A. (2003). Carcass composition and yield of versus 2001 broilers when fed representative 1957 and 2001 broiler diets. Poult. Sci..

[bib0010] Havenstein G.B., Ferket P.R., Qureshi M.A. (2003). Growth, livability, and feed conversion of 1957 versus 2001 broilers when fed representative 1957 and 2001 broiler diets. Poult. Sci..

[bib0011] Hosford A.D., Hergenreder J.E., Kim J.K., Baggerman J.O., Ribeiro F.R.B., Anderson M.J., Spivey K.S., Rounds W., Johnson B.J. (2015). Effects of supplemental lysine and methionine with zilpaterol hydrochloride on feedlot performance, carcass merit, and skeletal muscle fiber characteristics in finishing feedlot cattle. J. Anim. Sci..

[bib0012] Kastenschmidt J.M., Ellefsen K.L., Mannaa A.H., Giebel J.J., Yahia R., Ayer R.E., Pham P., Rios R., Vetrone S.A., Mozaffar T., Villalta S.A. (2019). QuantiMus: a machine learning-based approach for high precision analysis of skeletal muscle morphology. Front. Physiol..

[bib0013] Konnaris M.A., Brendel M., Fontana M.A., Otero M., Ivashkiv L.B., Wang F., Bell R.D (2022). Computational pathology for musculoskeletal conditions using machine learning: advances, trends, and challenges. Arthritis Res. Ther..

[bib0014] Kostrominova T.Y., Reiner D.S., Haas R.H., Ingermanson R., McDonough P.M. (2013). Automated methods for the analysis of skeletal muscle fiber size and metabolic type. Int. Rev. Cell Mol. Biol..

[bib0015] Legland D., Arganda-Carreras I., Andrey P. (2016). MorphoLibJ: integrated library and plugins for mathematical morphology with ImageJ. Bioinformatics.

[bib0016] Patael T., Piestun Y., Soffer A., Mordechay S., Yahav S., Velleman S.G., Halevy O. (2019). Early post hatch thermal stress causes long-term adverse effects on pectoralis muscle development in broilers. Poult. Sci..

[bib0017] Petracci M., Mudalal S., Soglia F., Cavani C. (2015). Meat quality in fast-growing broiler chickens. World's Poult. Sci. J..

[bib0019] Piestun Y., Halevy O., Shinder D., Ruzal M., Druyan S., Yahav S. (2011). Thermal manipulations during broiler embryogenesis improves post-hatch performance under hot conditions. J. Thermal Biol..

[bib0018] Piestun Y., Harel M., Barak M., Yahav S., Halevy O. (2009). Thermal manipulations in late-term chick embryos have immediate and longer-term effects on myoblast proliferation and skeletal muscle hypertrophy. J. Appl. Physiol..

[bib0020] Piestun Y., Patael T., Yahav S., Velleman S.G., Halevy O. (2017). Early post hatch thermal stress affects breast muscle development and satellite cell growth and characteristics in broilers. Poult. Sci..

[bib0021] Rahmati M., Rashno A. (2021). Automated image segmentation method to analyse skeletal-muscle cross section in exercise-induced regenerating myofibers. Sci. Rep..

[bib0022] Reyes-Fernandez P.C., Periou B., Decrouy X., Relaix F., Authier F.J. (2019). Automated image-analysis method for the quantification of fiber morphometry and fiber type population in human skeletal muscle. Skeletal Muscle.

[bib0023] Smith L.R., Barton E.R. (2014). SMASH – semi-automatic muscle analysis using segmentation of histology: a MATLAB application. Skeletal Muscle.

[bib0024] Soglia F., Petracci M., Davoli R., Zappaterra M. (2021). A critical review of the mechanisms involved in the occurrence of growth-related abnormalities affecting broiler chicken breast muscles. Poult. Sci..

[bib0025] Stringer C., Wang T., Michaelos M., Pachitariu M. (2021). Cellpose: a generalist algorithm for cellular segmentation. Nat. Methods.

[bib0026] Uni Z., Ferket P.R. (2003).

[bib0027] Wen Y., Murach K.A., Vechetti I.J., Fry C.S., Vickery C.D., Peterson C.A., McCarthy J.J., Campbell K.S. (2017). MyoVision: software for automated high-content analysis of skeletal muscle immunohistochemistry. J. Appl. Physiol..

[bib0028] Zuidhof M.J., Schneider B.L., Carney V.L., Korver D.R., Robinson F.E. (2014). Growth, efficiency, and yield of commercial broilers from 1957, 1978, and 2005. Poult. Sci..

